# From surfing to diving into the tumor microenvironment through multiparametric imaging mass cytometry

**DOI:** 10.3389/fimmu.2025.1544844

**Published:** 2025-04-11

**Authors:** Marco Erreni, Maria Rita Fumagalli, Matteo Marozzi, Roberto Leone, Raffaella Parente, Raffaella D’Anna, Andrea Doni

**Affiliations:** ^1^ Unit of Multiscale and Nanostructural Imaging, IRCCS Humanitas Research Hospital, Rozzano, Milan, Italy; ^2^ Department of Biomedical Sciences, Humanitas University, Pieve Emanuele, Milan, Italy

**Keywords:** imaging mass cytometry, tumor microenvironment, multiplexed histopathology, cancer, pancreatic cancer

## Abstract

The tumor microenvironment (TME) is a complex ecosystem where malignant and non-malignant cells cooperate and interact determining cancer progression. Cell abundance, phenotype and localization within the TME vary over tumor development and in response to therapeutic interventions. Therefore, increasing our knowledge of the spatiotemporal changes in the tumor ecosystem architecture is of importance to better understand the etiologic development of the neoplastic diseases. Imaging Mass Cytometry (IMC) represents the elective multiplexed imaging technology enabling the *in-situ* analysis of up to 43 different protein markers for in-depth phenotypic and spatial investigation of cells in their preserved microenvironment. IMC is currently applied in cancer research to define the composition of the cellular landscape and to identify biomarkers of predictive and prognostic significance with relevance in mechanisms of drug resistance. Herein, we describe the general principles and experimental workflow of IMC raising the informative potential in preclinical and clinical cancer research.

## Introduction

Cancer development and progression are regulated by a complex multistep process involving heterogeneous interacting components (1, 2). The biological and spatial relationship between the diversity of cells composing the tumor microenvironment (TME) plays a pivotal role in tumor progression and in response to therapies (3). Recent technological advances currently support cancer research. In particular, single-cell technologies have largely increased the knowledge on TME landscape, providing detailed proteomic and genomic profiles, and leading to the identification of new potential prognostic and therapeutic biomarkers (4–6). Most of these technologies make use of a suspension of cells collected from tumor specimens, thus lacking the spatial information of cell localization within tumor tissue. On the other hand, conventional tissue analysis technologies, such as IHC and immunofluorescence, lack multiplex capabilities, mainly due to limitations in the number of markers simultaneously visualized on the tissue (7). To overcome these limitations, several *in situ* imaging multiplex technologies have been applied to the study of TME (8), including fluorescence-based multiplexed techniques (9–13). However, most of these techniques involve multiple cycles of staining, acquisition and stripping, leading to possible modifications to the antibody-epitope affinity, damaging of the tissue architecture and requiring precise image co-registration processes for the analysis (10, 14–18). A different fluorescence-based solution has been recently provided by the Orion platform, who developed a one-shot immunofluorescence high-plex method, followed by Hematoxylin-Eosin imaging on the same tissue slide (19). However, the number of antibodies simultaneously analyzable is limited and a robust image post-processing is required to correct for system aberrations and separate the contribution of overlapping signals. In addition, tissue autofluorescence, especially in formalin-fixed paraffin-embedded (FFPE) tissue, represents a limitation in probe detection (20). Finally, IHC chromogens and fluorophores are often not chemically stable, thus limiting sample long-term storage.

Methods based on detection of elements and ions through mass spectrometry, relying on non-fluorescent signals, represent promising alternative technologies (21, 22). Among these mass spectrometry-based techniques, Imaging Mass Cytometry (IMC) (23), combines the multiplex capacity of mass cytometry (CyTOF, Standard Biotools^®^) with IHC and is successfully applied in preclinical and clinical cancer studies (24–28).

## Imaging combined with mass spectrometry: IMC

**Figure 1:**
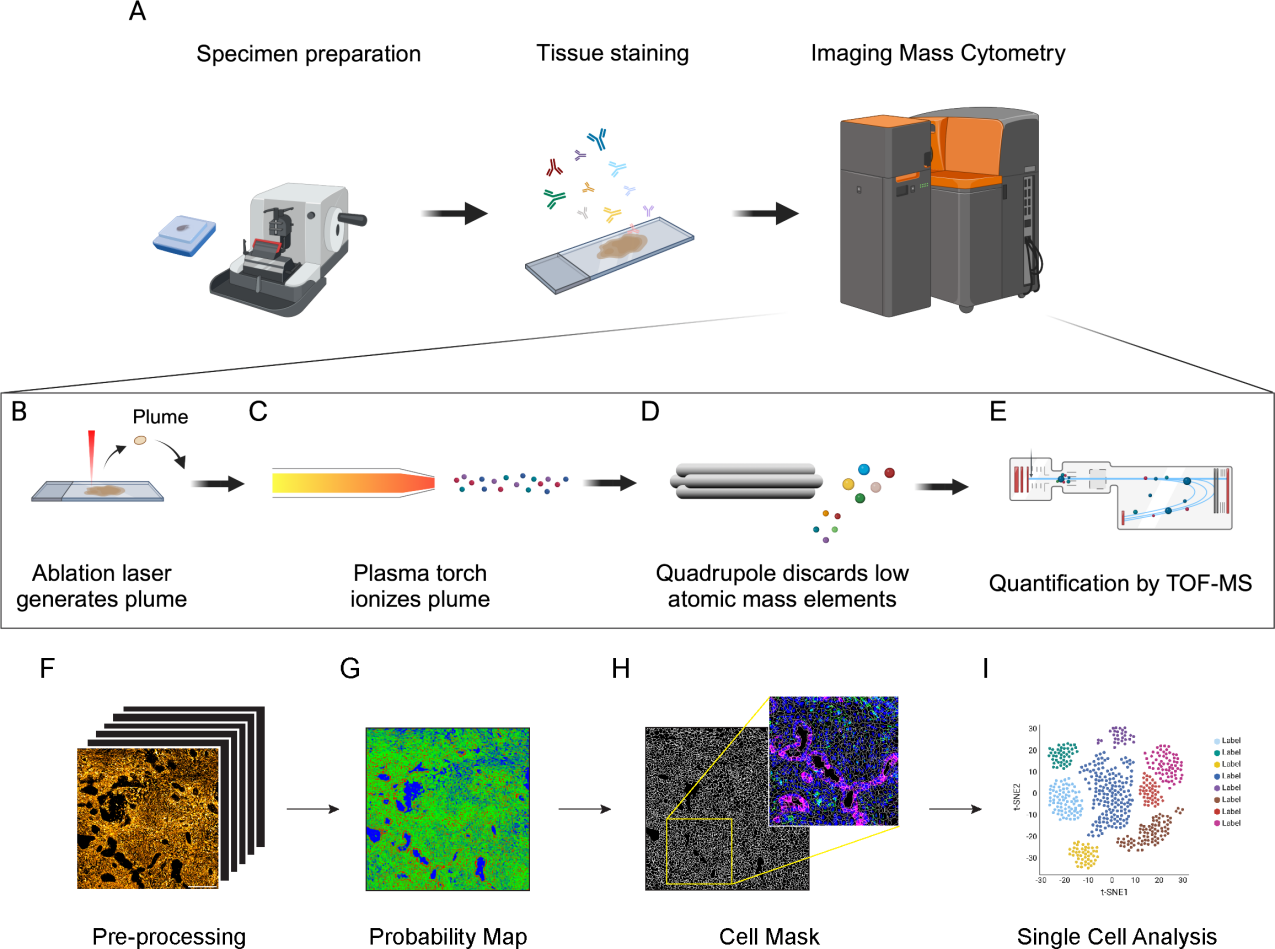
Schematic principle of Imaging Mass Cytometry (IMC) technology and downstream analysis. **(A)** Antibodies (up to 43) conjugated with metal isotopes are hybridized on tissue slides (FFPE or frozen) as done for conventional immunohistochemistry. Slides are then inserted in the IMC analyzer (Hyperion Imaging System, Standard Biotools®) for data acquisition. **(B)** Within the IMC analyzer, a UV laser with a 1μm^2^ beam spot ablates the tissue, generating a plume. **(C)** The plume is ionized by an inductively coupled plasma. **(D)** Ions are then filtered by a quadrupole mass spectrometer to discard elements with lower atomic mass. **(E)** Ions with high atomic mass are finally quantified by a Time-of-Flight (TOF) mass spectrometer (MS). **(F)** MCD files are converted into multi-channel and single-channel.tiff files. Image pre-processing is required to remove background noise and artifacts, including speckles and hot pixels. **(G)** Pixel classification is applied to IMC images to generate probability maps and distinguish cell nuclei (red), membrane/cytoplasm (green) and background (blue). Based on probability maps, watershed segmentation generates a cell mask for single-cell identification. The cell mask can then be overlaid on the original IMC signal to assure the accuracy of the segmentation process (inset, Blue: Nuclei; Green: CD45; Magenta: Pan-Cytokeratin). **(H)** Cell masks generated in the cell segmentation process are combined with raw.tiff files and exported as a single-cell file containing the signal intensity and spatial coordinates of each marker in each cell. **(I)** These data are then used for cell annotation and downstream analysis.

Differently from chromogen- and fluorescence-based technologies, IMC relies on the use of metal-tagged antibodies, allowing the simultaneous visualization of up to 43 different markers on the same tissue slide (23, 29–31). The staining protocol is based on conventional immunohistochemistry procedures and can be applied to both frozen and FFPE tissues (28, 32, 33) (**Figure 1A**). Subsequently, a laser system ablates the stained tissue with a spatial resolution of 1μm^2^ (**Figure 1A**). The ablated material is then collected by the CyTOF analyzer, where it is ionized through an inductively coupled plasma (ICP) (**Figures 1B, C**) and the metal masses are quantified by a time-of-flight (TOF) mass spectrometer (MS) (23, 34) (**Figures 1D, E**). Metals can be also linked to DNA probes for the detection of mRNA, combining transcriptomic and proteomic approaches (35). To date, 42 metals are commercially available for the antibody conjugation, in addition to a cation nucleic acid intercalator (191-193 Iridium), which is used to identify nuclei (Standard Biotools^®^) (31, 34). Further signal from other isotopes can be recorded during the acquisition as control channels for contaminants (e.g. Ar, Ba, Pb). Metal tags in the optimal detection range (141-176 A) have less than 4% of signal spillover, avoiding signal overlap (36, 37).

In addition to IMC, multiplexed ion beam imaging (MIBI) is another technology that takes advantage of metal-tagged antibodies (38, 39). MIBI is based on the principle of secondary ion mass spectrometry: briefly, by applying an O_2_^+^ duoplasmotron primary ion beam to the tissue slides, secondary ions are released from the tissue and directly introduced into a TOF-MS systems for the metal detection. A MIBI detection system collects not only metal ions from conjugated antibodies, but quantifies all the elements, including those naturally present in tissues, such as ^12^C and ^31^P, which can be used to infer the general structure of the tissue and the nuclei, or ^56^Fe, which has been correlated with the amount of heme-oxygenase-1 in spleen macrophages (39). Compared to IMC, that is destructive, MIBI has minimal effect on the tissue, allowing multiple rounds of acquisition of the same ROIs. In addition, the image resolution is adjustable by regulating the acquisition time. However, the multiplexing capacity of MIBI technology has often been limited to 7 channels acquired in a single round, with only recent advances improving it to 15-20 and up to 40 channels (40, 41).

## IMC images and single-cell profiles

During IMC acquisition, the system records, for each ablated tissue spot, the intensity of the signal (collected as dual counts) coming from metal-tag antibodies and the coordinates of the spot inside the ablated region of interest (ROI). Thus, ROIs can be visualized as multichannel images, where individual ablated spots are identified as a pixel, and the vector of metal-tag dual counts represents the pixel intensity for each channel. In this context, it would be straightforward to analyze IMC data applying traditional multiplexed immunofluorescence pipelines, but various technical issues, such as different signal intensity and spatial resolution, need to be addressed (42). As an example, data normalization and background removal are affected by the mean intensity value of IMC signals, that are often lower than a conventional fluorescence signal. Moreover, approaches based on a general simple analysis of the staining patterns and signal intensity would overlook a large fraction of the data complexity. To properly investigate the biological information provided by IMC acquisitions, it is necessary to set-up a workflow consisting of sequential steps of data pre-processing, cell segmentation (in order to convert pixel-based signal into single-cell data), cell annotation and downstream analysis (42). A variety of open-source libraries, algorithms and commercial software tools have been applied to IMC data: the majority of these tools addresses only a subset of the complete analytical workflow, while others encompass the full range of processes, from raw data extraction to downstream analysis (28, 43, 44).

Herein, we briefly describe the steps related to IMC analyses, highlighting some of the existing methods. A more comprehensive comparison of the analysis methods is reported in the following references (23, 42, 45).

### Pre-processing

IMC data are saved in .txt and MiniCAD Design file (MCD) format, which can be easily visualized as false-color multichannel images using the MCDviewer® software (Standard Biotools®). Data from .MCD files are generally converted into multi-channel .tiff/.ome.tiff files, suitable for downstream analyses, using either MCD viewer or dedicated python libraries (mcdparser and napari) (43). Alternatively, .txt files can be converted into multichannel .tiff images using more generic matrix-to-tiff libraries (**Figure 1F**). Although autofluorescence and signal overlap do not affect IMC images, artifacts and background noise can have an impact on the analysis. Hot pixels and speckles are the most common artifacts in IMC images, generally due to detector abnormalities and non-specific binding of aggregated antibodies or contaminants (46, 47). Artifacts can also arise due to signal spillover, when a channel signal is excessively high and affects the signal detection of neighboring channels. Background noise, mainly derived from the staining procedures and non-specific antibody binding, is often detectable and can be non-negligible for rare or low-expressed markers. Most of these issues can be minimized by a proper titration of the antibodies and an optimization of the staining protocol. In addition, signal amplification methods have been proposed for IMC applications in order to overcome issues related to low signal-to-noise ratio (48, 49). Several pre-processing methods have been generated to remove channel crosstalk, non-specific staining and aggregates, including MAUI, IMC-Denoise and Fiji (ImageJ) plugins (28, 46, 50–52).

### Cell segmentation

Cell segmentation is the most challenging and critical step in IMC procedure and hence the correct interpretation of the data directly depends on the accuracy of this process (53–55). In most of the cases, cell segmentation relies on mixed manual/automatic methods for pixel classification, where a model is trained to discriminate between nuclear, cytoplasm/membrane and background pixels, in order to generate a probability map (**Figure 1G**). Cell boundaries are then automatically identified from the probability map using thresholding-, watershed- or inference- based methods. The combination of Ilastik single-pixel classification (56) and CellProfiler segmentation (57) is a commonly used strategy for IMC data processing (28, 43, 53, 55). This method requires, for each single experiment and panel, the presence of operators with expertise both in histology, for the correct identification of cells and background, and in bioinformatics, to correctly train the models. To reduce the impact of operator-dependent classification, more advanced approaches, based on convolutional neural networks (CNN), have been developed, such as Dice-XMBD and YOUPI. These methods only require a single training on a set of images and can be applied to other experiments, independently from the antibody panel used (47, 58). Regardless of the implemented method, it is crucial to check the reliability of the cell segmentation process by overlaying cell masks with specific nuclear and cytoplasm/membrane staining, in order to assure the accuracy of the downstream analysis (**Figure 1H**). In densely packed tissues, the relatively low spatial resolution of IMC (1μm^2^/pixel) can be insufficient to properly discriminate cell boundaries. To overcome this limitation, some approaches combining fluorescence and IMC have been developed: for example, the MATISSE pipeline combines IMC-derived signal for cytoplasm/membrane and nuclear staining, together with fluorescence DAPI staining on the same tissue section (53). By acquiring the same region in fluorescence and IMC modalities, it is possible to overlay DAPI and Iridium signals, taking advantage of the higher resolution of the immunofluorescence signal to better discriminate packed cells, thus obtaining more accurate cell masks (53). Even if the cell segmentation process has been done flawlessly, lateral signal bleed-through can occur between cells in very close proximity, resulting in the generation of marker expression patterns that are biologically implausible, such as CD3/CD20 expressing lymphocytes, or CD3/PanCK expressing epithelial cells (42). A specific compensation algorithm named RedDSEA has been recently developed, to correctly assign IMC signal to the proper cell of origin (54).

### Data analysis

The process of cell segmentation results in the generation of a cell mask, where the signal intensity for each acquired channel, morphological parameter and tissue localization are associated to every single cell. Thus, each ROI is generally associated with data files (txt, csv, xls, etc.) containing per-cell information, that can be then used for cell annotation and downstream analysis, using essentially the same strategies developed for scRNA-seq and cytometry (**Figure 1I**). Cells with incoherent signals, area or debris should be discarded from the analysis at this step. Dimensional reduction strategies, such as t-SNE and UMAP (59, 60), are widely used for the 2D representation of these high-dimensional datasets, preserving as much as possible data structure. Cell annotation is usually performed by unsupervised clustering of cell analysis, allowing the unbiased identification of cell populations and the potential discovery of new cell phenotypes (42, 43, 61). Differently from scRNA-seq, where existing gene-sets can be used for the annotation of new experiments, the cellular subtypes can be defined in IMC experiments only on the base of detected channels, and the level of detail used to characterize the sub-populations depends on the chosen panel of markers. The main advantage of the IMC data is that the information on tissue localization of the cells and their spatial interaction is preserved. Thus, once a specific cell population has been detected, besides relative frequency estimate, it is possible to perform neighborhood analysis to identify preferred cell partnership, recognize spatial enrichment or avoidance between cell clusters, define spatial signatures involving different subtypes and determine a specific cell network (61–63).

For IMC downstream analysis, both packages and software not specifically developed for IMC analysis (such as Seurat, Histocat, QuPath) are currently used, with few precautions and adjustments, as well as more spatial-oriented pipelines such as Imacyte, Simpli or Spicyr, depending on the specific research questions (42, 44, 62–65).

In conclusion, while IMC data processing and analysis are still challenging for users with limited experience in image analysis and computational skills, the wide number of developed pipelines highlights the number of perspectives that can be applied and biological questions that can be addressed by IMC.

### Technical considerations

There are several aspects that need to be taken into consideration in the setup of an IMC experimental workflow. First, the selection of the markers included in the panel is crucial, since the information derived from the IMC analysis directly depend on this choice. A good balance between conventional markers able to identify macro-population, and markers selected to investigate specific biological questions is advisable. While the latter subset could be used to describe in detail the specific subpopulation of interest, the macro-population markers are intended to identify, theoretically, all the cellular compartments in the tissue, such as tumor cells, immune cells, stromal cells and endothelial cells, to correctly support the cell segmentation procedures (61). Alternatively, it is possible to use commercially available kits for cell segmentation, which can be combined with conventional IMC antibody panels, as well as broad spectrum membrane and pan-Actin antibodies (27, 31, 66). As the technology relies on the specificity and affinity of antibodies for their targets, antibody validation is strictly required before and after metal conjugation, since metal-tag can induce modifications and affect the antibody performance (36). Antibodies should be tested with conventional histological methods before and after metal-conjugation, to confirm the immunodetection pattern. The use of tissues of different origin, such as tonsils, lymph nodes as well as tumor tissues, could be helpful (31). After metal conjugation, antibodies need to be titrated to get the optimal signal-to-noise ratio. Antibody titration is also necessary to set up the right conditions of antibody saturation for the recognition of its target, and allow a semi-quantitative evaluation of the expression of the different markers within the tissue. Therefore, the optimization of antibody-panel is an expansive and time-consuming process, but necessary to obtain reliable data. Indeed, most of the studies reporting the application of IMC are provided with detailed lists of antibodies included in the validated panels, which is extremely helpful for the IMC community (31, 67, 68). Papers focusing on the different aspects of the antibody validation process have been published and libraries of validated tissue-specific panels have been recently made available to the scientific community (69–71). The selection of the ROIs to acquire represents another crucial step to be considered. Generally, IMC images are small (in the range of 1-1.5mm^2^) compared to conventional entire tissue sections, so that the acquisition of multiple ROIs within the same tissue slice is mandatory. The number of ROIs to acquire per sample strictly depends on the type of tissue and its heterogeneity: the most rigorous methods for an appropriate sampling strategy is generally based on the acquisition of a matched whole tissue slide, stained with conventional histological methods, that can be used as a reference sample. In addition, several statistical methods have been developed to determine the best sampling strategies, optimizing both the number and the size of the ROIs to acquire, according to the tissue structure and the segregation level of specific cell types (72–74). Alternatively, tissue microarrays (TMAs), in which punches of tumors from different patients are selected and arrayed on the same slide, represent an interesting option to analyze large cohort of patients in a reasonable time and with affordable costs, since they can be processed and imaged together (75, 76).

## IMC in preclinical cancer studies

Preclinical mouse models of cancer have been widely used to recapitulate human disease and to investigate the complex biological processes occurring in tumor development and therapeutic responses (77, 78). Although IMC has been largely applied to analyze the TME in humans, only few studies have been published on mouse models of carcinogenesis (**Table 1**). Among them, Glasson and colleagues, set up a 31-antibody panel for the IMC investigation of FFPE mouse models of cancer (79). By applying IMC analysis, they characterized the tissue architecture and cell composition of B16-K1 melanoma and *Apc^Δ14+^* intestinal models of carcinogenesis. In particular, in the B16-K1 model, IMC analysis showed a preferential localization of T cells at the periphery of the tumor mass, while macrophages were able to infiltrate the tumor core, thus describing a feature that is typical of immune-excluded tumors (79). Similarly, Van Maldegem et al. developed a 27-antibody panel for frozen tissue of *K-RAS* mutated mouse model of lung tumors (80). They analyzed the TME of mouse lungs, providing quantitative information about the phenotype and spatial relationship of stromal and immune infiltrating cells, and how the inhibition of KRAS G12C promotes remodeling towards an enhanced immune activation state (80).

**Table 1:** Summary of the studies using IMC to investigate the TME.

Author	Research	Target Tumor	Reference
Glasson et al	Preclinical	Melanoma and *Apc^Δ14+^* intestinal model	(79)
Van Maldegem et al	Preclinical	Lung cancer	(80)
Zabransky et al	Preclinical	Hepatocellular carcinoma	(81)
Peran et al	Preclinical	Pancreatic cancer	(82)
Erreni et al	Preclinical	Pancreatic cancer	(28)
Strittmatter et al	Preclinical	Ovarian, lung and colon cancer	(85)
Strittmatter et al	Preclinical	Pancreatic cancer	(86)
Chang et al	Preclinical	Pancreatic cancer	(87)
Shen et al	Clinical	Hepatocellular carcinoma	(92)
Ravi et al	Clinical	Glioblastomas	(93)
Colombo et al	Clinical	B-cell lymphomas	(94)
Rigamonti et al	Clinical	Non-small cell lung carcinoma	(27)
Sorin et al	Clinical	Lung cancer	(75)
Jackson et al	Clinical	Breast cancer	(76)
Ali et al	Clinical	Breast cancer	(95)
Danenberg et al	Clinical	Breast cancer	(96)
Rogenes et al	Clinical	Breast cancer	(31)
Tornaas	Clinical	Head and neck squamous cell carcinoma	(99)
Xiang et al	Clinical	Lung squamous cell carcinoma	(100)
Cords et al	Clinical	Breast cancer	(101)
Cords et al	Clinical	Non-small cell lung cancer	(68)
Elyada et al	Clinical	Pancreatic cancer	(105)
Sussman et al	Clinical	Pancreatic cancer	(106)
Erreni et al	Clinical	Pancreatic cancer	(61)
Oetjen et al	Clinical	Myelodysplastic syndrome	(116)
Li et al	Clinical	Lung squamous cell carcinoma	(91)
Zhang et al	Clinical	Colorectal cancer	(117)
Bertocchi et al	Clinical	Colorectal cancer; liver metastasis	(120)
Fischer et al	Clinical	Breast cancer; lymph node metastasis	(123)
Kuett et al	Clinical	Breast cancer; bone, soft tissue, liver and brain metastasis	(124)
Hoch et al	Clinical	Metastatic melanoma	(125)
Martinez-Morilla et al	Clinical	Melanoma	(126)
Le Noac'h et al	Clinical	Small cell lung cancer	(127)
Hiltbrunner et al	Clinical	Non-small cell lung cancer	(128)
Bortolomeazzi et al	Clinical	Colorectal cancer	(129)
Mi et al	Clinical	Hepatocellular carcinoma	(66)
Carvajal-Hausdorf et al	Clinical	Breast cancer	(130)
Wang et al	Clinical	Breast cancer	(131)
Cao et al	Clinical	Gastric cancer	(132)
Cao et al	Clinical	Colorectal cancer	(133)

In addition to the description of the TME composition, IMC has been used to analyze the response to treatment in preclinical models. In syngenic models of hepatocellular carcinoma, IMC has been used to demonstrate the role of the TME in response to treatment with anti-PD-1 antibodies: interestingly, the infiltration of tumor-associated macrophages (TAMs) with an M2-phenotype and the interaction between T cells and cancer-associated fibroblasts (CAFs) were associated with the resistance to treatment (81). Similarly, in a mouse model of pancreatic cancer, it has been shown that the expression of Cadherin-11 was associated with CAF pro-tumorigenic activity and that the administration of anti-Cadherin-11 antibody was able to inhibit this effect by decreasing the infiltration of FoxP3^+^ T cells in the tumor (82). Recently, our group validated a 28-marker panel to investigate the TME in orthotopic and genetic models of pancreatic ductal adenocarcinoma (PDAC) (28). We compared the KPC model, expressing mutant isoforms of *KRAS* and *TP53* genes in pancreatic cells (83), and the Panc02-cell orthotopic transplanted model (84). While in the Panc02 model stromal cells surrounded the dense neoplastic mass, in the KPC model the desmoplastic stroma infiltrated the tumor and it is characterized by the presence of cells expressing markers of CAFs, such as αSMA, Vimentin and Desmin. In addition, markers included in the panel allowed to discriminate KPC tumors having different grade of desmoplasia. Distribution of blood vessels was different too: while in the orthotopic model CD31^+^ blood vessels mainly surround the tumor mass, in the KPC model blood vessels infiltrate the tumors, better resembling the features of human PDAC (28). Moreover, the composition and localization of immune infiltrating cells were different between the models. In the orthotopic model, the tumor core was mainly infiltrated by TAMs and CD8^+^ T cells. Differently, a spatial association of CD8^+^ T cells, CD4^+^ T cells, neutrophils, dendritic cells and TAMs was identified at the tumor-stromal interface and in the KPC model (28). These differences could be due to the diverse nature of the immune response, with the orthotopic model characterized by a more acute inflammatory microenvironment compared to a mild, chronic inflammation in the genetic model.

IMC has also been applied in studies aimed at comparing drug delivery methods and pharmacodynamics. Strittmatter and colleagues applied IMC, in combination with mass spectrometry imaging, to analyze the biodistribution of an aurora kinase B inhibitor (AZD2811) in patient-derived xenograft models of ovarian, lung and colon cancer (85). By using a 27-antibody panel, they described the cell composition and distribution in the TME. Subsequently, they applied mass spectrometry imaging to visualize and quantify the distribution of AZD2811-loaded nanoparticles. Finally, by combining these techniques, they demonstrated an accumulation of the drug in macrophage-rich regions. Similarly, the same group analyzed the distribution and the effect of gemcitabine and its metabolites in the KPC model of pancreatic cancer, revealing that gemcitabine metabolites were able to induce DNA damage in regions characterized by a high proliferation rate (86).

Since IMC detects metal isotopes, it can be used to monitor the biodistribution of platinum-based therapies. In pancreatic cancer patient-derived xenograft models, Chang and colleagues combined the detection of platinum and a 14-antibody panel to evaluate the accumulation of cisplatin in collagen-rich areas of the tumor (87).

## IMC in clinical cancer studies

**Figure 2:**
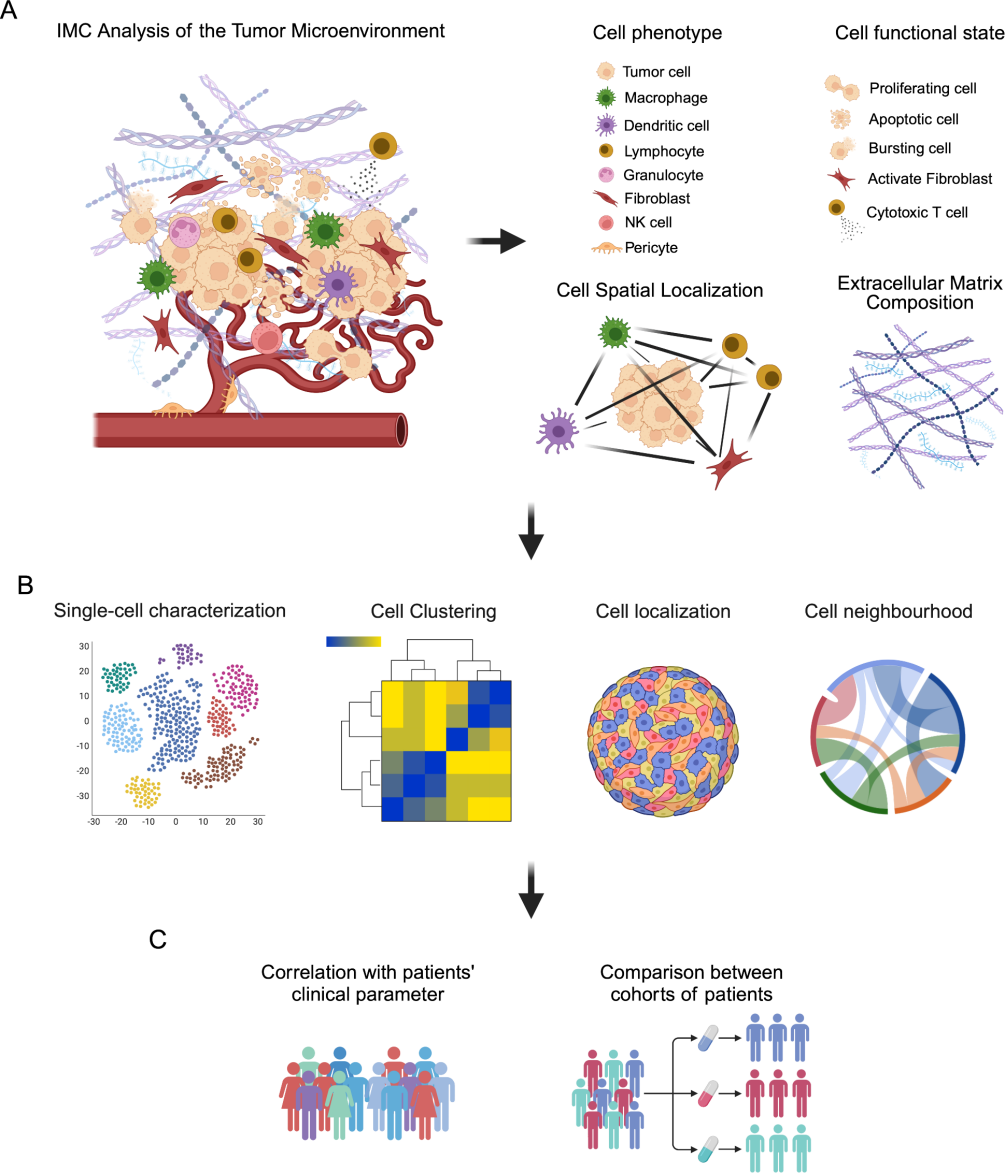
Schematic representation of IMC analysis of the tumor microenvironment (TME). **(A)** TME is a complex ecosystem where cells of different phenotypes, such as tumor cells, immune cells, stromal cells and endothelial cells, immerse in a reactive extracellular matrix, cooperate to influence tumor progression and response to therapeutic intervention. IMC analysis provide information related to the phenotype of cells, their functional state and their localization in the TME, as well as the composition of the extracellular matrix. **(B)** These information can be analyzed by single-cell visualization with dimensional reduction strategies, such as t-SNE and UMAP, and cell clustering, as well as by investigating cell localization and their spatial relationship (neighborhood analysis) in the TME. **(C)** These data can be then correlated with patients’ clinical parameter and used to compare different cohorts of patients to find markers and signatures able to predict patients’ outcome and prognosis.

IMC has been widely applied to investigate the composition of TME, the identification of new biomarkers and the effectiveness of therapeutic approaches in several studies on human cancer (25, 88, 89). As previously discussed, IMC allows to visualize up to 43 different markers on the same tissue slide, providing not only qualitative but also quantitative information about the amount of different metal-conjugated antibodies in the corresponding tissue spot. The combination of multiple antibodies is necessary to adequately identify different cellular phenotypes and their state of activation, as well as the spatial relationship of different cell populations within tumor, immune and stromal compartments (**Figure 2A**).

These aspects make IMC particularly appropriate for the in-depth, single-cell analysis of cancer tissues and for the correlation between imaging data with patients’ clinical and pathological features (**Figures 2B, C**).

### Profiling the cellularity of the tumor microenvironment

Several studies reported the feasibility of IMC to describe the complexity of the TME in different cancer subtypes and for the quantification of specific cellular markers (**Table 1**) (33, 90, 91). Shen and colleagues applied IMC for the investigation of TME in human hepatocellular carcinoma (HCC): they quantified 36 biomarkers in a cohort of 134 HCC patients and 7 healthy donors and identified three major types of intratumor regions, characterized by a distinct distribution patterns of cancer, stromal and immune cells. In addition, the analysis of the cellular neighborhood showed that different types of cells were spatially associated to form regional functional units, which are relevant to patients’ clinical outcomes (92). Similarly, Ravi et al. provided a detailed description of the TME in glioblastomas, confirming its pivotal role in tumor development (93). In another study on diffuse large B-cell lymphomas (DLBCL), IMC has been used to characterize tumor and immune cell architecture, in correlation with clinicopathological features. The data showed that, instead of being histo-pathologically monotonous, DLBCL displays a complex tumor architecture and that modification in tumor topology can be associated with clinically relevant features (94). More recently, Rigamonti and colleagues combined artificial intelligence (AI)-aided histopathology with IMC to investigate the microenvironment of non-small cell lung carcinoma (NSCLC) (27). Specifically, an AI-based approach was applied to hematoxylin and eosin (H&E) stained NSCLC tissues to identify tumor cells and generate a classifier of neoplastic cell spatial clustering. Then, consecutive sections were used for the IMC analysis of 24 markers related to tumor, stromal and immune cell populations and immune activation, resulting in the identification of 11 macrophage clusters and T cells with different tissue localization. Combining AI-powered histopathology and IMC, the authors provided insights into NSCLC microenvironment and used the data to translate tumor characteristics into a classifier capable to predict patients’ prognosis and response to therapy (27). Still in the context of lung cancer, Sorin and colleagues applied IMC to investigate the tumor and immunological landscape in a large cohort of lung adenocarcinoma patients (75). Using deep learning, the authors were able to predict patients at higher risk of progression after surgery, which could be extremely useful for clinical management after surgical resection (75).

In breast cancer, IMC approach allowed the identification of multiple cellular phenotypes in the TME, providing a refined histopathological classification of tissue samples (76). In addition, single-cell spatial analysis described cellular inter- and intra-tumor heterogeneity, resulting in the identification of novel subtypes of breast cancer associated with distinct clinical outcome (76). The same group applied IMC on tissues from a genetically well-characterized cohort of breast cancer patients from the Molecular Taxonomy of Breast Cancer International Consortium (METABRIC) (95). They found that genomic diversities correspond to differences in tumor and stromal cell phenotypes, showing how genomes shape the composition and the architecture of TME in breast cancer. Also in this cohort of patients, IMC data revealed that cell phenotype and cellular neighborhood were associated with patients’ prognosis (95). Similarly, Danenberg and colleagues demonstrated the presence of different structures of TME in breast cancer subtypes showing association with somatic alterations and genomic profiles (96). These structures are characterized by enrichment for *CASP8* and *BRCA1* mutations and are associated with poor prognosis in estrogen-receptor positive breast cancer (96).

Several studies demonstrated the pivotal role of CAFs in shaping the tumor microenvironment (97, 98). CAF-focused IMC panels have been validated in the context of breast cancer and head and neck squamous cell carcinoma (HNSCC), resulting in the identification of distinct CAF subtypes, expressing different levels of αSMA and FAP (Fibroblast Activation Protein) (31, 99). Xiang and colleagues described a specific interaction between CAFs and monocytic myeloid cells in the TME of lung squamous carcinoma, highlighting the role of CAFs in regulating monocyte recruitment and differentiation (100). More recently, Cords and colleagues combined single-cell RNA sequencing and IMC to identify nine CAF subtypes and one pericyte population in human breast cancer (101). In particular, IMC analysis provided information about the spatial distribution of CAFs in the TME and their relationship with tumor and stromal cells, vessels and classes of neighboring cells. Moreover, they proposed a general classification system for CAFs that can be useful for their identification and functional description across different cancer types (101). The same group analyzed the CAF population in a large cohort of NSCLC, identifying 11 CAF phenotypes and demonstrating that CAFs represent an independent prognostic factor for NSCLC patients’ survival. In addition, they showed that the CAF phenotype influenced the TME composition, regulating inflammation and immune cell infiltration (68). In PDAC, CAFs play a pivotal role in shaping TME (102). Although IMC has been applied to study the composition of pancreatic islet in both type 1 and type 2 diabetes (103, 104), only a limited number of studies have employed IMC for this purpose. In PDAC, IMC was used to show the presence of a novel identified CAF subtype, defined as antigen-presenting CAFs (apCAFs), which expresses MHC-II and CD74, but is negative for the classical co-stimulatory molecules (105). They further demonstrated that apCAFs are able to activate CD4^+^ T cells, confirming their immune-modulatory capabilities. In addition, Sussman and colleagues identified a specific subset of CD68^+^/CD44^+^/HLA-DR^Low^ macrophages located within the vascular niche and not spatially associated with T and B cells, suggesting a unique pro-angiogenic activity different from the other, more abundant, antigen-presenting cells in the PDAC microenvironment (106). More recently, our group applied a 31-antibody panel to investigate the cellular composition of TME in 8 PDAC patients (61). We defined 19 different subpopulations of CAFs having distinct phenotype, tissue localization and spatial relationship with other cells in PDAC TME (61). Beside the already described myCAFs and apCAFs, we identified a subpopulation of podoplanin^+^/cadherin-11^+^ CAFs, which were associated with higher levels of carbohydrate antigen 19-9 (CA19-9), shorter disease-free survival (DFS) and overall survival (OS). In addition, we found a spatial association of podoplanin-expressing CAFs with CD4^+^ T cells and CD44^+^ macrophages, suggesting a role in the modulation of immune response. Moreover, we identified 4 distinct CAF subtypes expressing FAP, that were specifically enriched in regions of tumor-stroma interface and associated with tumor cells and CD44^+^ macrophages. This observation suggested the presence of an extracellular matrix remodeling niche, that sustains tumor cell invasion and promotes PDAC progression. Of note, FAP^+^ CAFs were also associated with higher levels of CA19-9 (61). Moreover, we identified 7 different types of tumor cell subtypes, characterized by the differential expression of markers associated with disease progression, invasion and resistance to therapy, including carbonic anhydrase IX (CA-IX), S100A4 and CD44 (61, 107–109). Moreover, we identified a specific subtype of tumor cells expressing PTX3, a molecule already associated with tumor progression in several types of cancer (110–115), in patients having distant metastasis at the time of the diagnosis.

As mentioned earlier, the possibility to combine multiple markers in the same tissue slide provides the identification of new complex cell phenotypes. Oetjen and colleagues described a new population of erythroid cells expressing CD71, CD235a and high levels of the proliferative marker Ki-67 in erythroid islands in normal bone marrow samples and myelodysplastic syndromes (116). In lung squamous cell carcinoma, a new subpopulation of CD3^-^/CD4^+^ cells, characterized by the high expression of FoxP3 and TNFα, has been identified, suggesting their proinflammatory role in the tumor immune microenvironment (91). Similarly, in colorectal cancer, IMC allowed the spatial identification of an abnormal EpCAM^+^/CD4^+^ T cell population, which also expressed CCR5, CCR6 and increased levels of phospho-p38 MAPK and phospho-MAPKAPK2 (117).

It has been shown that in the metastatic process, the primary tumor can affect the metastatic target organ to form a pre-metastatic niche that supports cancer cell metastasis and growth (118, 119). Bertocchi and colleagues applied IMC to analyze the pre-metastatic microenvironment in colorectal cancer liver metastasis (120). They found that following damage to the gut vascular barrier (121, 122), C17 *Escherichia Coli* colonizes the liver and generates a microenvironment that is suitable for colorectal cancer cell metastasis. In particular, they combined IMC with fluorescence *in situ* hybridization (FISH) and RNA sequencing, showing that bacteria preferentially localized close to SOX9^+^ cancer cells, both in primary and liver metastatic tumors (120). In breast cancer, Fischer and colleagues compared the single-cell phenotypes of primary tumors and matched lymph node metastases (123). They observed a phenotypic diversity between primary tumor and metastatic lymph node and identified single-cell phenotypes of tumor cells, prone to disseminate, that are associated with patients’ prognosis (123). More recently, Kuett and colleagues assembled three different antibody panels (75 markers) to study the tumor and immune cell composition of primary and metastatic breast cancer tissues (124). Interestingly, they found that the proportion of the same tumor cell phenotypes differs between primary tumors and matched metastatic tissues. In addition, immune cell infiltration is generally reduced in the metastatic sites, showing a higher proportion of remodeling myeloid cells, as well as cytotoxic and exhausted T cells (124).

In conclusion, IMC provided the possibility to deeply describe the composition of TME, leading to the identification of phenotypically and spatially distinct new rare cell subpopulations, that can be potentially used as innovative prognostic and therapeutic markers.

### Correlating IMC data with disease’s outcome

In addition to the description of the complexity of the tumor microenvironment and the identification of new potential biomarkers, IMC has been successfully applied to evaluate patients’ responses to anti-cancer treatments. Hoch and colleagues demonstrated that, in metastatic melanoma, chemokines CXCL9 and CXCL10 were associated with CXCL13^+^ exhausted T cells, suggesting their role in recruiting B cells and in the formation of tertiary lymphoid structures (TLS). In addition, TLS showed a spatial enrichment of naïve and naïve-like T cells, which are involved in anti-tumor immunity and are predictive of response to immune checkpoint blockade (125). Similarly, IMC has been applied to correlate the composition of TME and the survival of melanoma patients treated with immunotherapy, pointing out some potential predictive biomarkers, such as beta2-microglobulin (126). In small-lung cancer, Le Noac’h and colleagues used IMC to identify predictive biomarkers to stratify patients who could benefit from the combination of chemotherapy and anti-PD-L1 immunotherapy. They found that higher infiltration of CD4^+^/CD8^+^ and regulatory T cells was associated with longer progression-free survival (127). More recently, Hiltbrunner and colleagues investigated the mechanism responsible for the acquired resistance to immune-checkpoint inhibitor (ICI) therapy in patients with NSCLC: IMC analysis revealed that in resistant neoplastic lesions, despite the broadly distributed infiltration, T cells co-expressed a variety of immune checkpoints and immune modulatory enzymes, resulting in an exhausted T cell phenotype having limited effector functions that, in turn, can be responsible for the lack of response to ICI therapy (128). In colorectal cancer (CRC), response to immune checkpoint blockade is variable. Bortolomeazzi and colleagues applied IMC to demonstrate that, in hypermutated CRC, anti-PD1 drugs released the PD1-PD-L1 interaction between macrophages and CD8^+^ T cells, finally promoting cytotoxic antitumor activity (129). In a recent study on hepatocellular carcinoma, IMC analysis showed that, in patients treated with a combination of cabozantinib and nivolumab, the spatial interaction between CD8^+^ T cells and Arginase 1-expressing macrophages represents a key feature of the TME in non-responders. In addition, an interaction network between macrophage-enriched and lymphocyte-enriched areas with tumor regions was observed in non-responders (66). Carvajal-Hausdord and colleagues applied IMC to analyze tumor tissues in trastuzumab-treated breast cancer patients: they found that the expression of HER2-extracellular segment (HER2-ECD) was reduced in relapsed patients. Moreover, authors found a correlation between high expression of HER2-ECD and cytotoxic T cell response: this observation could explain the better prognosis of trastuzumab-treated patients showing high tumor-infiltrating lymphocytes (130). More recently, Wang and colleagues analyzed the effect of immune checkpoint blockade (ICB) in patients with triple-negative breast cancer (TNBC) (131). IMC analyses on 42 markers revealed that CD8^+^ TCF1^+^ T cells and MHC-II^+^ cancer cells are predictors of response to ICB treatment. In addition, responsive tumors are highly infiltrated by granzyme B^+^ T cells, while in resistant tumors cancer cells are CD15-positive (131).

As already described in the preclinical model, IMC can be used to monitor the biodistribution of platinum-based therapies in both tumor and non-tumor tissues. In gastric cancer, it has been shown that platinum was detectable up to 72 days after preoperative chemotherapy in resected surgical samples and its concentration correlated with an increased pathological response. In addition, platinum was strongly associated with collagen-rich regions, thus explaining the variability in platinum concentration in tumor tissues among patients (132). The same group also showed that in colorectal cancer patients treated with FOLFOX, platinum was detectable in skin biopsies more than 60 months after the completion of the therapy, providing a possible explanation for the oxaliplatin-induced peripheral sensory neuropathy, observed as an adverse effect of the treatment (133).

In summary, IMC analysis can provide important information related to the effect of therapeutic interventions on the tumor microenvironment. Due to the heterogeneity of cancer, the selection of the right treatment plans and the assessment of efficacy responsiveness are of primary importance for cancer patients. In this context, IMC provides a deep single-cell analysis of the phenotypes and interactions of cells in TME, promoting the tumor pathological classification, the prediction of the response to therapeutic treatments and the assessment of the risk of patients’ relapse.

## Future direction and perspectives

In the last decade, the field of multiplex imaging, and in particular IMC, has grown rapidly and the number of available protocols, designed panels and papers applying IMC to address biological questions is constantly increasing. In this review, we provided several examples of the contribution of IMC in preclinical and clinical studies in the field of oncological research. With the possibility to analyze multiple markers on the same tissue section, avoiding the technical issues associated to fluorescence-based multiplexed methods, IMC allows the identification of cells with different phenotypes, as well as their localization and spatial interaction in the TME, and to correlate this information with the risk of tumor progression, the efficacy of therapeutic interventions and, finally, patients’ prognosis. The technology is still evolving, with the introduction of new features at both hardware and software level, such as the recently introduced possibility to acquired whole slides sections, sampling the tissue with a lower spatial resolution. Although this approach prevents the possibility of a single-cell analysis, it allows a more comprehensive distribution of markers in the tissue section, performing a spatial clustering of the targeted proteins that could serve as the bases for ROI positioning in the subsequent analyses at single-cell levels. In addition, a new methodology based on X-ray fluorescence imaging technology has been recently proposed for the detection of lanthanide metal-conjugated antibodies (134): this approach has the advantage to preserve the integrity of the sample and can be extended to 3D imaging, but it currently shows lower sensitivity compared to IMC and requires the use of a synchrotron (134). Another direction for the development of IMC is represented by its application in 3D, that would dramatically improve the analysis of spatial processes, including tumor angiogenesis and cancer cell invasion (135).

Multi-omics technologies are rapidly developing (136) and the integration of genomic and mass spectrometry imaging data with IMC analysis would allow to obtain and correlate complementary information, to go even deeper in the characterization of the TME and to identify signatures capable to predict tumor progression and response to therapy. In addition, IMC analysis can be used to validate, at proteomic level, data related to the transcriptional regulation of genes in the TME. To this aim, efforts have been made to increase the spatial resolution of spatial transcriptomic and mass spectrometry imaging towards the single-cell (137–139). In addition, computational protocols and algorithms able to integrate different types of datasets derived from multi-omics technology are necessary (140, 141).

In conclusion, IMC-based studies significantly contribute to unveil the complexity of TME and to discover new predictive signatures of tumor progression and mechanisms of resistance to therapy. Currently, IMC is mainly applied for research purposes. To integrate this technology into clinical practice, efforts are needed to standardize staining procedures and data analyses, and to harmonize data, to ensure the generation of reproducible and reliable information that can be used for the development of effective diagnostic and therapeutic approaches for precision medicine.
